# Possible nanoantenna control of chlorophyll dynamics for bioinspired photovoltaics

**DOI:** 10.1038/s41598-019-43545-4

**Published:** 2019-05-09

**Authors:** Sergey V. Gaponenko, Pierre-Michel Adam, Dmitry V. Guzatov, Alina O. Muravitskaya

**Affiliations:** 1 B. I. Stepanov Institute of Physics, National Academy of Sciences, Minsk, 220072 Belarus; 2 Light, nanomaterials and nanotechnologies (L2n), University of Technology of Troyes, 12 rue Marie Curie, 10004 Troyes, France; 3 Yanka Kupala State University of Grodno, Grodno, 230023 Belarus

**Keywords:** Nanoparticles, Silicon photonics

## Abstract

In the context of using portions of a photosynthetic apparatus of green plants and photosynthesizing bacteria in bioinspired photovoltaic systems, we consider possible control of the chlorophyll excited state decay rate using nanoantennas in the form of a single metal and semiconductor nanoparticle. Since chlorophyll luminescence competes with electron delivery for chemical reactions chain and also to an external circuit, we examine possible *excited state decay inhibition* contrary to *radiative rate enhancement*. Both metal and semiconductor nanoparticles enable inhibition of radiative decay rate by one order of the magnitude as compared to that in vacuum, whereas a metal nanosphere cannot perform the overall decay inhibition since slowing down of radiative decay occurs only along with the similar growth of its nonradiative counterpart whereas a semiconductor nanoantenna is lossless. Additionally, at normal orientation of the emitter dipole moment to a nanoparticle surface, a silicon nanoparticle promotes enhancement of radiative decay by one order of the magnitude within the whole visible range. Our results can be used for other photochemical or photovoltaic processes, and strong radiative decay enhancement found for dielectric nanoantennas paves the way to radiative decays and light emitters engineering without non-radiative losses.

## Introduction

### Chlorophyll photoluminescence in photosynthetic bacteria and plants

Photosynthesis is the major process on the Earth producing organic matter from inorganic precursors promoted by light absorption and involving multiple stages like energy transfer, electron transfer, relaxation processes, the *chlorophyll reaction center* being at the focus of the process and chlorophyll combined with carotenoids in spatially organized complexes serving as light harvesting and energy transfer counterparts^1,2^. The overall scenario from a photon absorption by a light harvesting complex to an electron release by a reaction center for further chemical processing takes less than 100 ps and has a quantum yield close to 1 (i. e., nearly every photon absorbed finally results in an electron delivery to the chemical chain)^3^. Photophysical aspects of photosynthesis basically end at the stage of an electron release by a reaction center for further involvement in chemical processes. A chlorophyll molecule in the reaction center can be excited either directly by absorbing red or blue light itself or by energy transfer from other chlorophyll molecules and/or from carotenoids absorbing the green light. One more function of carotenoids is modulation (control) of the reaction center efficacy by means of the so-called *non-photochemical quenching* of chlorophyll photoluminescence (i. e., providing a controllable non-radiative path of the chlorophyll excited state relaxation to shunt an electron delivery to chemical stages of photosynthesis)^4,5^. This non-photochemical quenching enables not only plant survival under conditions of ultrastrong illumination but also helps to modulate photosynthesis rate under condition of other threats, e. g., water shortage. At the same time low but non-zero green light absorption by chlorophyll molecules is supposed to enable chlorophyll in deep leaf tissues to be used for light harvesting under condition of low illumination levels^6^. The above interplay of photostimulated processes in chlorophyll and carotenoids represent an example of the multivariant interactions in green leaves in the course of photosynthesis^7^.

Excited electron states of a chlorophyll molecule have actually the *three paths* in the photosynthesis scenario. *The first*, and the principal one, is the release of the high-energy electron to the chemical processing chain. *The second* is the radiative decay with intrinsic lifetime of a few nanoseconds. *The third* one is the nonradiative, probably both intrinsic and extrinsic decays influenced by the ambient medium polarity, temperature and tentatively, by the carotenoids-generated impact, to adjust the electron release rate in accordance with volatile illumination level and other external conditions. In a solution, molecular chlorophyll features the quantum yield up to 35% in polar solvents like water, alcohols, amines^8,9^ and much lower values in nonpolar solvents whereas in green leaves it varies from 5% to 1% with temperature rise from 77 K to 275 K^10^.

Chlorophyll photoluminescence does not bear significance as a stage or a process directly participating in or contributing to photosynthesis. Instead, it can be purposefully used only as a signature to monitor the rate of photosynthesis. High photoluminescence was found to typically correlate with lower photosynthesis yield and *vice versa*. This important correlation discovered in 1989^11^ is widely applied for *in vivo* photosynthesis studies in plants^12–14^.

### Plasmonics and luminescence: Quenching and enhancement interplay

Plasmonics is known to deal with *enhanced light—matter interaction* resulting from high local concentration of electromagnetic radiation in the sub-wavelength vicinity of metal nanobodies, their junctions or assemblies, the so-called “hot spots” develop nearby and therein. There are actually 3 processes modified by the proximity of a metal nanobody to a quantum system under consideration (an atom, a molecule, or a tiny piece of solid like a *quantum dot*). These are (i) enhanced excitation rate resulting from the incident light local concentration; (ii) modified (enhanced or inhibited) radiative decay rate, often referred to as the photon local density of states (LDOS) effect; (iii) modified non-radiative decay rate owing to energy transfer to a metal nanobody resulting in heating. Additionally, under condition of strong light—matter coupling, excitonic plasmon-polaritons can be generated (so-called “plexcitons”)^15,16^ enabling light energy transfer through a metal-dielectric system mediated by electronic excitation in the matter nearby. Since strong light—matter coupling needs special and rather tough conditions, in what follows it will be left beyond consideration.

In a typical plasmonic experiment with fluorescing species, experimenters tend to make use of the positive balance of the above 3 processes. The most typical plasmonics application to photoluminescence is referred to as *metal enhanced fluorescence (MEF)*^17,18^ which implies an arrangement enabling domination of metal enhanced incident light intensity over the loss in quantum yield *Q* resulting from non-radiative decay enhancement. For a perfect emitter with *Q* = 1, a non-radiative decay channel promoted by a metal proximity will always result in lower *Q*. The situation resembles to large extent the effect of an *antenna* in radiophysics thus proving the emerging conception of *nanoantennas* in nano-optics^19^. However, similar to radiophysical antennas, proximity of a metal body offers an option to substantially increase the intrinsic efficiency of an emitter with considerable internal losses, i. e., in the optical language, with *Q* ≪ 1. This may result in the overall photoluminescence intensity enhancement over several orders of the magnitude for poor emitters^20^ and, what is extremely essential in optoelectronics, even in enhancement of the efficiency of electrically pumped light-emitting devices, semiconductor LEDs or organic ones, OLEDs, where making use of the incident intensity enhancement is not the case at all^21,22^.

For molecular species involved in photosynthesis, plasmonic effects have been experimentally shown to increase light absorption^23^ and fluorescence^24–29^ in the context of bioinspired photovoltaics^30^, i. e., to use delivery of an electron from a chlorophyll reaction center to the outer electric circuit. However, in the context of the above discussion, enhancement of chlorophyll photoluminescence cannot directly assist in higher photocurrent generation. Moreover, metal-enhanced fluorescence typically occurs along with enhancement of excited state decay rate thus bypassing electron transfer into an external circuit. It seems also that many experiments on plasmonic improvement of photovoltaic devices were not successful because of the enhanced recombination rate preventing efficient charge separation in a device. Accordingly, the early consideration of plasmonic effects in photochemistry suggested photostability improvement owing to a fast bypass created for an excited state by a metal proximity^31^. Nowadays, e. g., semiconductor quantum dots and proteins have been found to feature higher photostability in presence of metal nanobodies^32,33^.

### Plasmonics for photovoltaics: Why inhibition of decay?

For the both photochemical reactions and photovoltaic devices the *long-lived* excited states are favorable since long lasting electron excited states raise up a probability for an excited electron to be involved in the other processes rather than to return radiatively or non-radiatively to the ground state. Is there a break for optical nanoantennas to bioinspired photovoltaic components which rely on implementation of photosynthetic fragments? The above discussion shows that it is *inhibition* of excited state decay which could improve performance of bioinspired photosynthetic photovoltaic cells. The intrinsic non-radiative decay process can hardly be altered by nanophotonic approaches whereas the radiative decay does depend upon the properties of the local environment not only in the context of a solution polarity but also being fundamentally dependent on the photon local density of states (LDOS) which is defined by a nanoantenna. According to the Barnett—Loudon sum rule^34^, for a given point in space, enhancement (inhibition) of the radiative decay rate of a quantum emitter as compared to that in vacuum will necessary be compensated by the opposite changes otherwise so that the overall modification of radiative lifetimes over the wide frequency spectrum will always tend to zero. This fundamental property has been qualitatively confirmed both theoretically and experimentally for microcavities and photonic crystals (see Chapter 14 in^35^ and refs therein for detail). The similar phenomenon of coexisting radiative rate enhancement and inhibition should occur in metal and dielectric nanoantennas but it seems that inhibition of radiative decay with nanoantennas remained beyond systematical analysis except for the early work^36^ where preliminary analysis was made within an electrostatic approximation for which application to bigger nanobodies is questionable. In the experiments, inhibition of radiative decay was not purposefully pursued for typical light emitters^19–22,37–39^.

In this work, we consider a number of simple cases which are feasible with metal and semiconductor nanoparticles and show that semiconductor antennas rather than metal ones promise noticeable inhibition of the excited state decay for an emitter in the wide spectral range embracing the chlorophyll luminescence spectrum of approximately 600–800 nm, and at the same time feature noticeable enhancement depending on emitter dipole moment orientation. We use the Mie theory^40^ throughout enabling reliable predictions for wide range of nanoparticle sizes as well as accounting for possible manifestation of magnetic properties in non-magnetic dielectric spheres.

## Results and Discussion

### Metal nanoantennas

**Figure 1:**
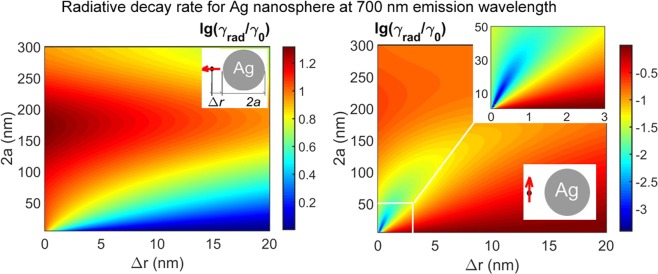
Calculated radiative decay rate at emission wavelength 700 nm for a silver solid sphere versus sphere diameter 2*a* and sphere-emitter distance Δ*r* for (left) normal and (right) tangential orientation of an emitter dipole moment. Ambient medium refraction index is 1.

**Figure 2:**
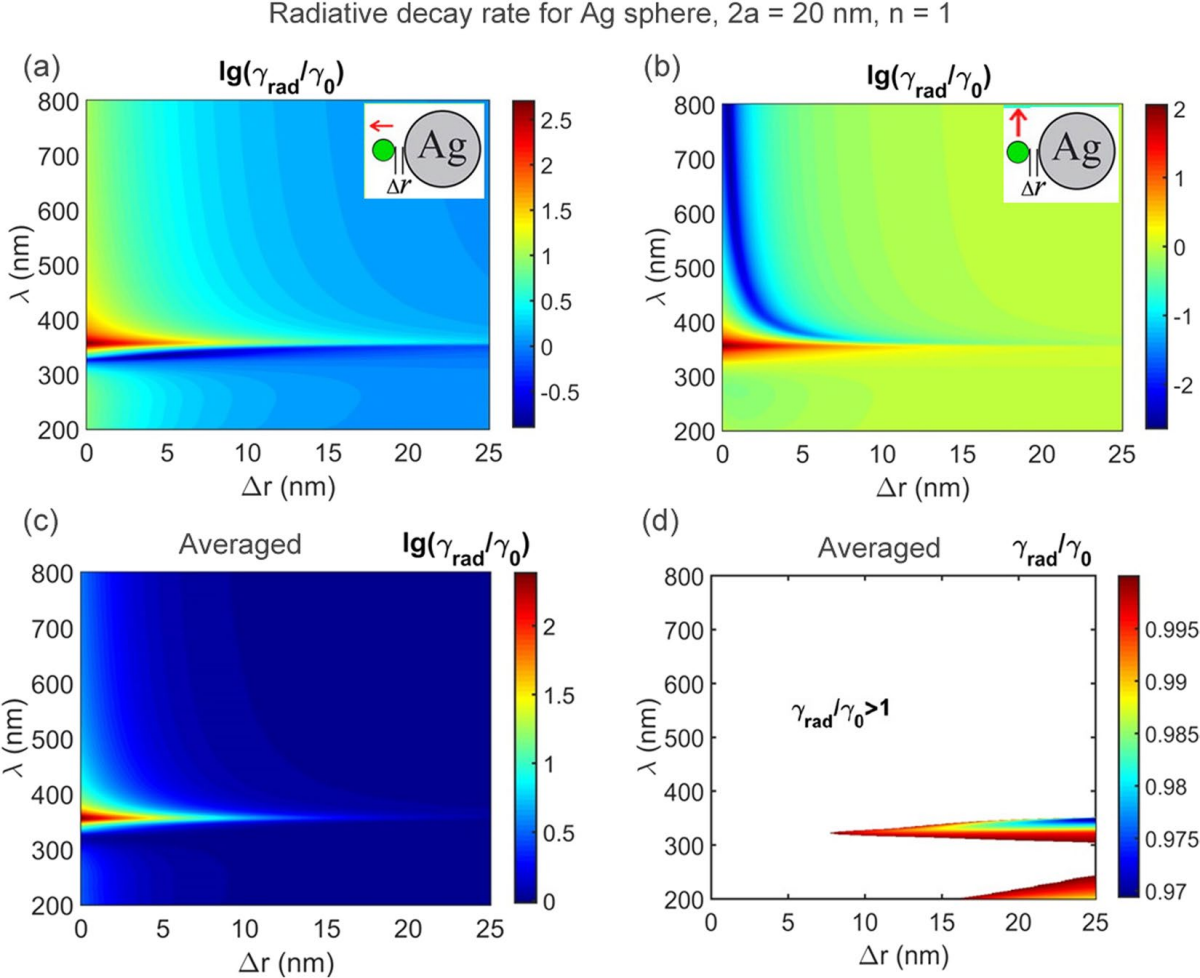
Calculated modification of radiative decay rate *γ*_rad_ with respect to vacuum rate *γ*_0_ for a dipole near a 20 nm Ag nanosphere versus emission wavelength λ and emitter – metal spacing Δ*r*. Ambient medium refractive index *n* = 1. (**a**) Normal dipole orientation; (**b**) tangential dipole orientation; (**c**) averaged over dipole orientation; (**d**) averaged over dipole orientation with extended *γ*_rad_/*γ*_0_ scale, color area shows inhibition (*γ*_rad_/*γ*_0_ < 1) whereas white area shows enhancement (*γ*_rad_/*γ*_0_ > 1) of the radiative decay rate. Note the linear scale *γ*_rad_/*γ*_0_ in (**d**) and the logarithmic scale otherwise. Calculations for 50 nm Ag spheres (Fig. 3) show nearly the same results though inhibition is slightly less (2 orders of the magnitude) than for 20 nm spheres.

**Figure 3:**
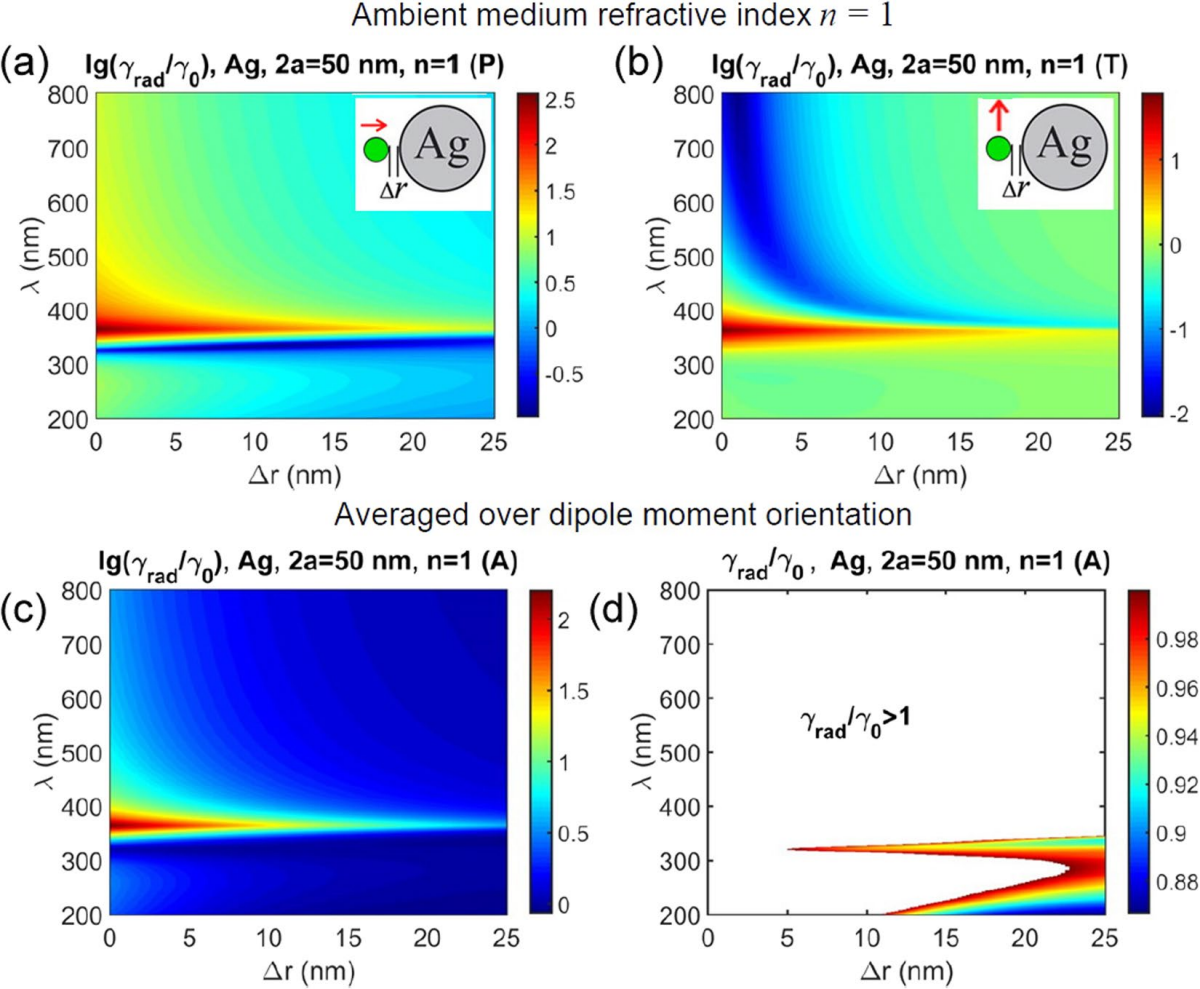
Calculated modification of radiative decay rate *γ*_rad_ with respect to vacuum rate *γ*_0_ for a dipole near a Ag nanosphere versus emission wavelength λ and emitter – metal spacing Δ*r*. Ambient medium refractive index *n* = 1. (**a**) Normal dipole orientation; (**b**) tangential dipole orientation; (**c**) averaged over dipole orientation; (d) averaged over dipole orientation with extended linear *γ*_rad_/*γ*_0_ scale, color area shows inhibition (*γ*_rad_/*γ*_0_ < 1) whereas the white area shows enhancement (*γ*_rad_/*γ*_0_ > 1) of the radiative decay rate. Note logarithmic scale lg(*γ*_rad_/*γ*_0_) in (**a**–**c**) and linear scale *γ*_rad_/*γ*_0_ in (**d**).

We present results for silver nanospheres as it was found to give promising effect in the spectral range of interest (600–750 nm). Gold nanoparticles were found to be less efficient. The nanoantenna effect dramatically depends on an emitter dipole orientation. Figure 1 shows radiative decay rate *γ*_*rad*_ modification with respect to the vacuum rate *γ*_0_ for a representative emission wavelength 700 nm. Normal orientation gives radiative decay enhancement whereas tangential orientation gives inhibition. A pronounced inhibition maximum about 10^3^ times occurs for tiny particles (30 nm or less) at very short distances about 1 nm. In what follows we consider the two representative cases of 2*a* = 20 nm and 2*a* = 50 nm (Figs 2 and 3).

Figure 2 summarizes calculations for a Ag 20 nm sphere. One can see, normal orientation (Fig. 2a) gives inhibited decay in the short-wave range with respect to the extinction maximum (about 350 nm for Ag solid sphere diameter 20 nm), and decay enhancement otherwise. Maximal radiative decay enhancement exceeds 2 orders of the magnitude. Inhibition occurs only in the narrow short-wave range (320–330 nm approximately) which is of no use for chlorophyll. Considerable inhibition of radiative decay in the spectral range of interest (600–800 nm) can be obtained for the tangential dipole orientation (Fig. 2b), the decrease in the radiative rate measuring more than 2 orders of the magnitude at a distance of 1–2 nm. However it was found that random orientation does not promise inhibition of the decay at all as is seen in Fig. 2c,d where calculation with averaging over dipole orientation are presented. Only in the narrow range in the near UV the ratio of *γ*_rad_/*γ*_0_ becomes just slightly less than 1.

**Figure 4:**
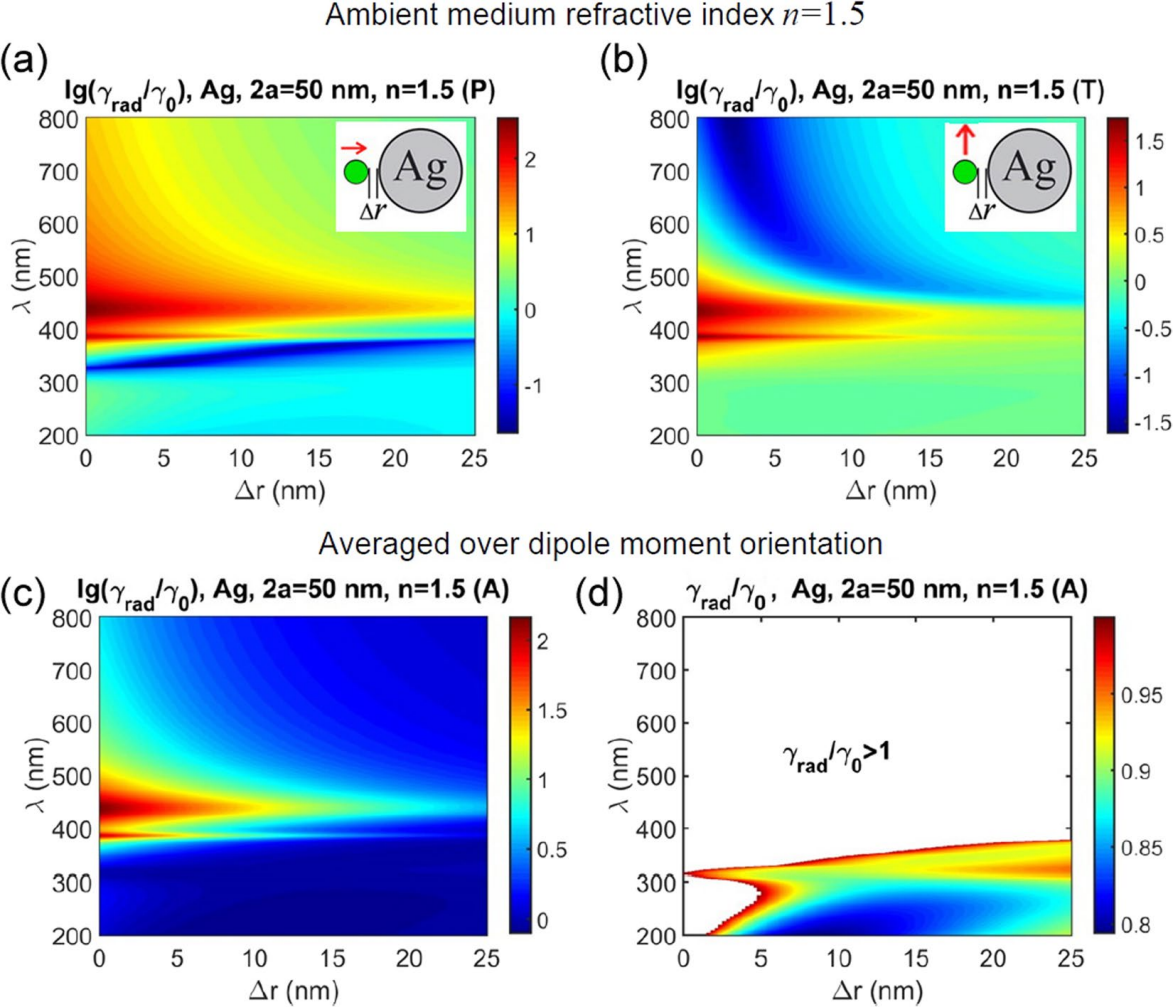
Calculated modification of radiative decay rate *γ*_rad_ with respect to vacuum rate *γ*_0_ for a dipole near a Ag nanosphere versus emission wavelength λ and emitter – metal spacing Δ*r*. Ambient medium refractive index *n* = 1.5. (**a**) Normal dipole orientation; (**b**) tangential dipole orientation; (**c**) averaged over dipole orientation; (d) averaged over dipole orientation with extended *γ*_rad_/*γ*_0_ scale, color area shows inhibition (*γ*_rad_/*γ*_0_ < 1) whereas white area shows enhancement (*γ*_rad_/*γ*_0_ > 1) of the radiative decay rate. Note the linear scale *γ*_rad_/*γ*_0_ in (**d**) and the logarithmic scale otherwise.

Ambient medium refractive index *n* was found to have no essential effect on the radiative decay properties. Figure 4 shows the same calculations as Fig. 3 but for *n* = 1.5. One can see, the main difference from the case *n* = 1 is a long-wave shift of the enhancement factor spectra which is generally very important for metal-enhanced luminescence experiments. However radiative decay inhibition remains to occur approximately in the same spectral ranges as for *n* = 1, though being less pronounced than in Fig. 1.

**Figure 5:**
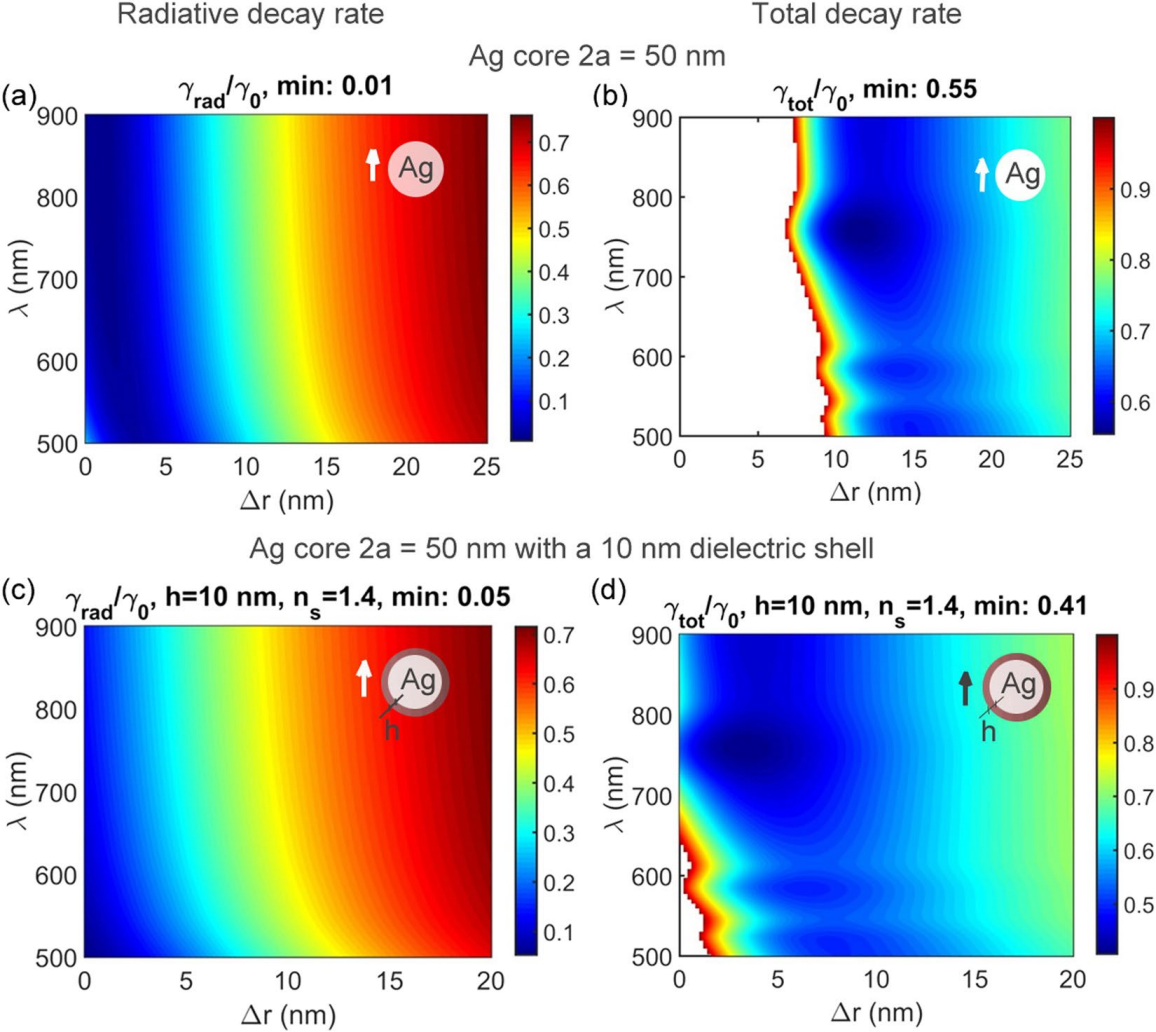
Calculated decay rates for tangential dipole orientation for a 2*a* = 50 nm Ag solid sphere versus emission wavelength λ and spacing Δ*r*. (**a**) Radiative decay rate; (**b**) Total decay rate; (**c**,**d**) show the same as (**a**,**b**) but for an extra *h* = 10 nm dielectric shell with *n*_*s*_ = 1.4. Ambient refractive index *n* = 1. Note linear scale everywhere. White areas correspond to decay rate enhancement. For the core-shell case the Δ*r* is a distance from a dipole to the outer shell surface, i.e., Δ*r* = *r*_0_ − (*h* + *a*).

Preferable tangential orientation imposes a restriction for experimental implementation such as, e. g., using chemical linkage of a molecule to metal surface^41^. The more dramatic restriction is that a metal nanoantenna inevitably brings *non-radiative* losses. Therefore, even if the conditions for inhibited radiative decay are met, enhanced non-radiative decay rate *γ*_*nonrad*_ can result in the overall total decay rate (${\gamma }_{{tot}}/{\gamma }_{0}=({\gamma }_{{rad}}+{\gamma }_{{nonrad}})/{\gamma }_{0}$) enhancement which is by no means desirable in the context of photovoltaic applications. Figure 5(a,b) allow for the contribution from non-radiative rate enhancement to be evaluated. The minimal radiative decay factor measures 0.01 whereas the total decay modification factor *γ*_*tot*_/*γ*_0_ is considerably bigger measuring only 0.55.

The next possible step in variation of experimental realization is using a dielectric shell over a solid metal sphere. In this case we found that inhibition of radiative decay is less pronounced (Fig. 5c) but because of slower non-radiative rate the overall decay rate can be lowered by a factor of 2.5 (Fig. 5d). Note that keeping on tangential orientation of an emitter is still a must.

Thus one can see that metal nanoparticles can promote enhanced decay rate more readily than inhibiton of decay. Inhibited decay is noticeable at fixed dipole orientation only. We consider this results can be useful in the context of plasmonic effects applied to photodetectors and also to photovoltaiс structures^42^ including recent proposal of possible electron photoemission^43–45^. It should be noted that dominating enhancement of decay (no matter whether it is radiative, non-radiative or mixed involving both radiative and non-radiative decay channels) promoted by metal nanobodies may deteriorate the overall performance of a photosensitive device even in case when plasmonic effects will enhance light energy absorption and photoelectron emission.

### Dielectric nanoantenna

The early hint to remarkable property of spontaneous decay modification by a dielectric nanobody can be found in the paper by Chew in 1987^46^. Nowadays, Krasnok and co-workers ingeniously suggested that entirely dielectric antennas including simple solid spheres can have the similar effect on light emission as the metal ones but without non-desirable non-radiative losses^47^. A single silicon nanosphere has been suggested as a starting design for the visible based on Si high refractive index *n* > 3 whereas absorptive losses are low owing to indirect interband transitions up to photon energies in the violet range (about 400 nm). The conception of a dielectric nanoantenna has gained the close consideration last years^48–51^. For the infrared spectral range from 1 to 2 µm, radiative decay rate has been calculated showing both enhanced and inhibited decay under certain conditons^52,53^. First experimental evidence of enhanced luminescence using entirely dielectric antennas has been reported^54^. An emitter decay rate when being placed inside a solid dielectric sphere (say, like a molecule in a polymer bead) was also found to be essentially modified^55^.

**Figure 6:**
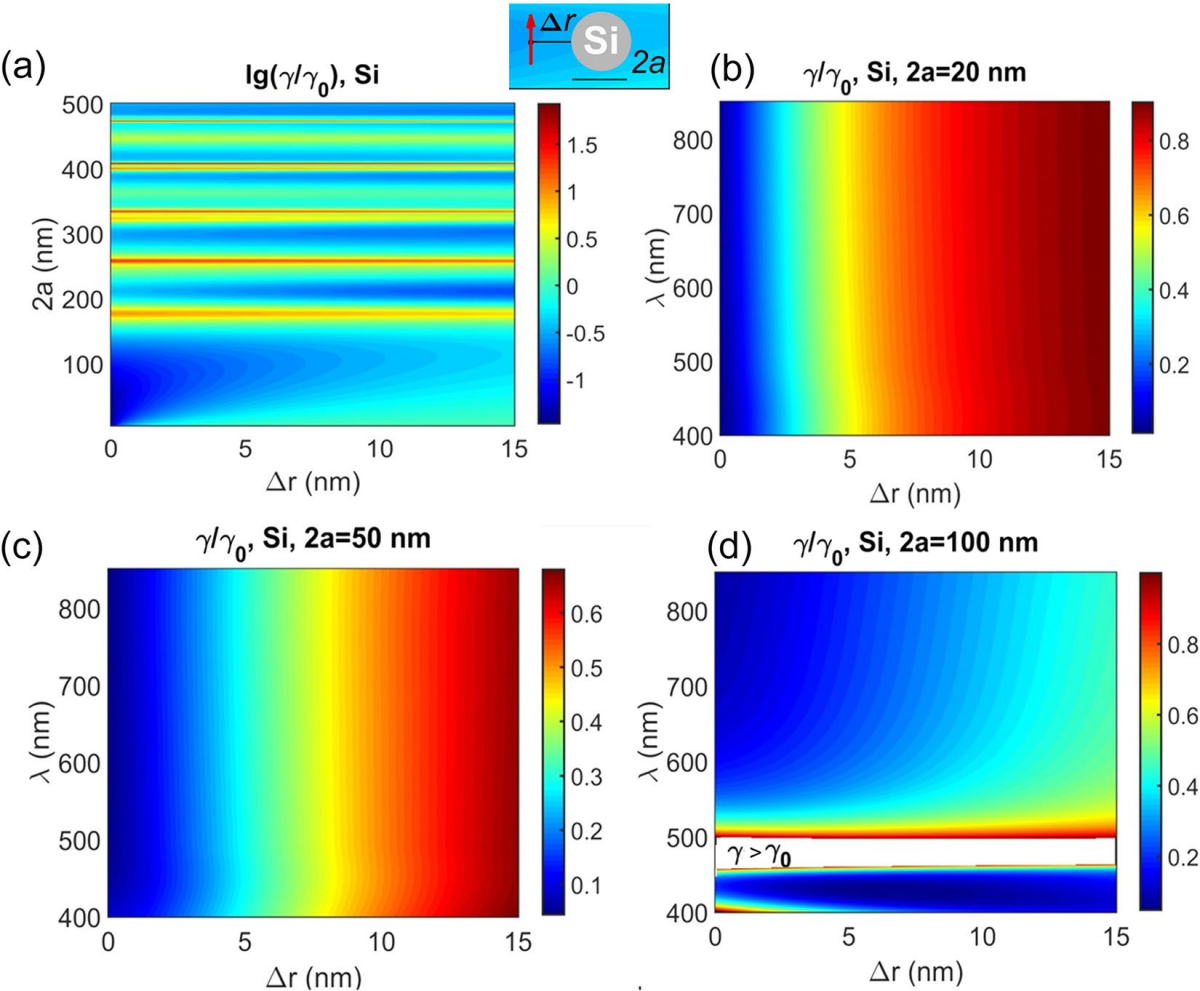
Calculated decay rate *γ*/*γ*_0_ for a dipole emitter near a Si solid sphere. (**a**) *γ*/*γ*_0_ as a function of sphere diameter 2*a* and emitter–sphere spacing Δ*r*, emission wavelength in vacuum being *λ* = 700 nm; (**b**–**d**) *γ*/*γ*_0_ as a function of emission wavelength in vacuum *λ* and emitter–sphere spacing Δ*r* for different 2*a* = 20, 50 and 100 nm, respectively. Dipole orientation is tangential as is shown in the insert. Absorptive losses are neglected. Ambient medium refractive index is *n* = 1. A white band in (**d**) corresponds to *γ*/*γ*_0_ > 1. Note logarithmic scale in (**a**) and the linear one otherwise.

**Figure 7:**
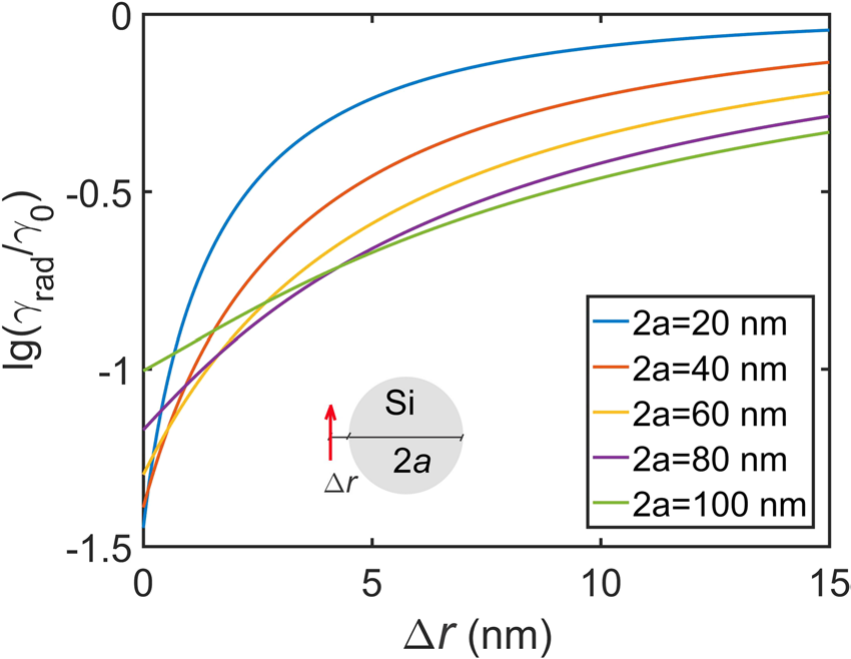
Calculated decay rate for emission wavelength in vacuum 700 nm for a dipole emitter near a Si solid sphere for the sphere diameters 2*a* being equal to 20, 40, 60, 80, and 100 nm. Dipole moment is parallel to the sphere surface. Ambient environment refractive index is *n* = 1.

We found that Si nanospheres with diameter 100 nm or less do promise more than one order of the magnitude slowing down in the decay rate (Fig. 6). Bigger spheres feature strong enhancement resonances from the whispering gallery modes (Fig. 6a). The slowing down occurs for an emitter dipole moment parallel to a Si particle surface (the tangential case).The effect of size is generally not pronounced for diameter less than 100 nm (Fig. 7) though smaller size tend to feature bigger range of decay modification, i. e., stronger effect at close distance and smaller effect at longer distance as compared to bigger particles. Slowing down depends on the emitter—sphere distance vanishing further than 15 nm and rising up to 10 times and more at zero distance. Remarkably, unlike metal nanoparticles, here the slowing down effect does not depend on emission wavelength in the spectral range examined.

**Figure 8:**
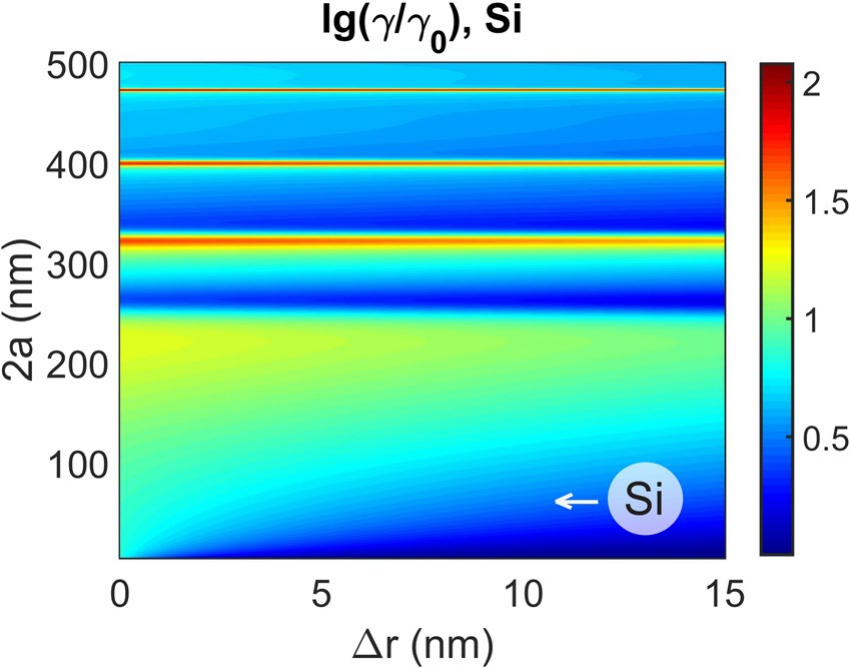
Calculated decay rate lg(*γ*/*γ*_0_) near a Si solid sphere versus sphere diameter 2*a* and emitter-sphere spacing Δ*r* for dipole orientation normally to the sphere surface.

**Figure 9:**
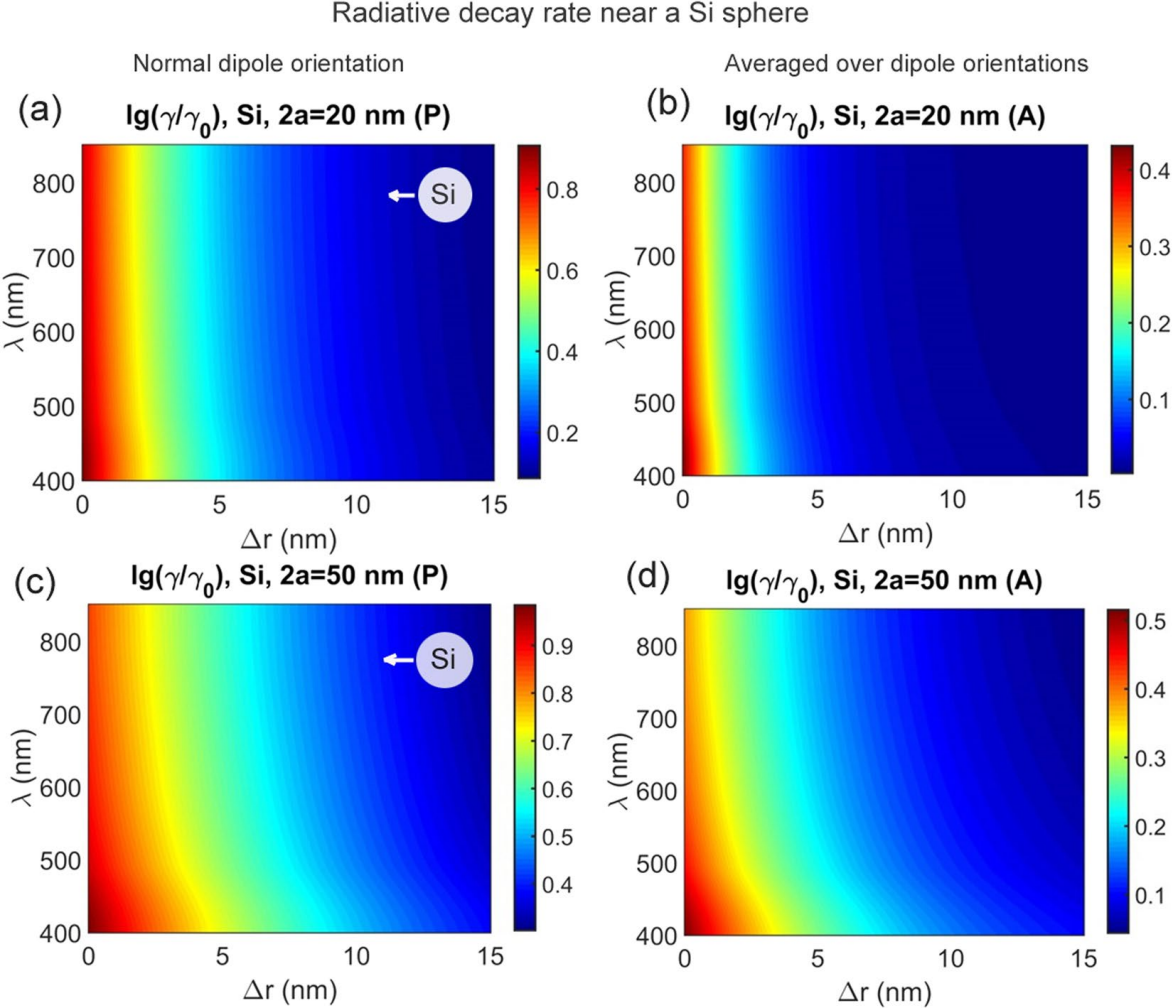
Calculated decay rate lg(*γ*/*γ*_0_) near a Si solid sphere. (**a**,**c**) Data for an emitter with dipole moment oriented normally to the Si sphere surface; (**b**,**d**) averaged over emitter orientation. Sphere diameters are 20 and 50 nm is indicated. Ambient medium refractive index is *n* = 1.

At normal orientation of an emitter dipole moment with respect to a sphere surface, Si spheres were found to enhance radiative (i.e. full) decay by one order of the magnitude in the wide spectral range and for the wide range of emitter—spacer distances. Sample results for this case are given in Figs 8 and 9. Note that enhancement grows for shorter wavelengths in correlation with Si refractive index. Since radiative decay enhancement for dielectric antennas has not been widely explored, these finding may stimulate experimental implementations of enhanced spontaneous emission which is on demand for light-emitting structures including LEDs and OLEDs. However in the context of photochemistry and photovoltaics where slowing down of decay is wanted these results mean that the tangetial dipole orientation should preferably be fixed by the proper adsorption, surface functionalization or chemical linking techniques.

Dipole orientation can be independently monitored by Raman signal enhancement studies. Photon LDOS equally affect spontaneous emission and scattering of light^56^. Therefore dipole orientation of a chlorophyll molecule can be monitored by surface enhanced Raman scattering (SERS) using Raman signatures of certain bonds. Namely, the bonds oriented parallel to the dipole moment of the target electronic transition will experience minimal SERS factors in the same experimental configuration.

To gain more insight in the origin of the inhibited spontaneous decay, we examine the angular diagrams of emitted power and trace changes in the diagram pattern as well as in radiation power for a dipole emitter near a spherical dielectric antenna. It is known that the rate of spontaneous transitions can be calculated as^35,57^

1 $$\frac{{\gamma }_{{rad}}}{{\gamma }_{0}}=\frac{{P}_{{rad}}}{{P}_{0}}$$

with *P*_0_ being the radiation power in vacuum, and *P*_rad_ being the power emitted by a dipole when located at the point of interest. The radiation power can be found by integration of the normal component of Poynting vector over the surface embracing the emitting system. The ratio in Eq.(1) can be presented as

2 $$\frac{{\gamma }_{{rad}}}{{\gamma }_{0}}={\int }_{4\pi }D({\rm{\Omega }})d{\rm{\Omega }},$$

with $d{\rm{\Omega }}=\,\sin \,\theta d\phi d\theta$ being an elementary solid angle. The *D*(Ω) in its turn^58,59^ can be calculated as described in Methods.

**Figure 10:**
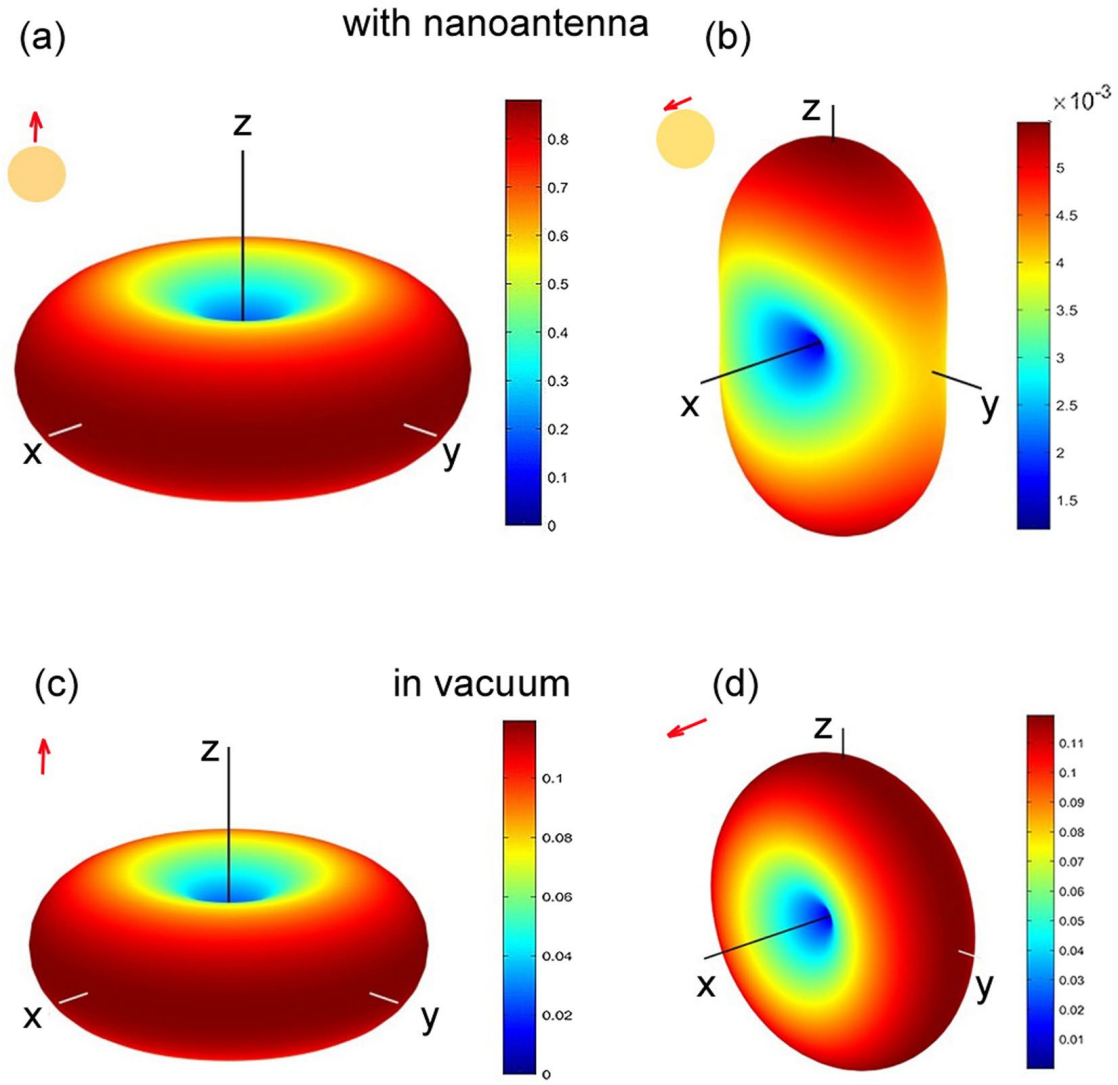
Calculated radiation patterns for a dipole (**a**,**b**) near a dielectric nanoantenna, and (**c**,**d**) in vacuum for dipole orientation along the z-axis (**a**,**c**) and along the x-axis (**c**,**d**). Note the factor of 10^−3^ in the (b) panel.

The results are presented in Fig. 10 for the two orientations of a dipole and compared with the same values for vacuum. One can see that in vacuum the pattern follows the rotation of a dipole in space, and the absolute emitted power is one order of the magnitude lower than for the case of a nanoantenna in Fig. 10(a) with a dipole oriented normally to its surface. At the same time for in-plane (tangential) dipole orientation (Fig. 10(b)), its emitted power falls by one order of the magnitude with respect to vacuum, and the pattern reshapes substantially. It can be treated as a consequence of a molecule interacting with its image, i. e., with induced dipole moment in the nanosphere.

There are commercially available oxide spherical beads (e.g. TiO_2_) but refractive index of oxides are low as compared to silicon. At first glance, spherical silicon nanoantenna looks like a useful computational model rather than a really feasible nanoobject. However there are certain approaches that can fabricate nearly spherical silicon nanoparticles by laser printing^60^ and colloidal^61^ techniques. Yet another reasonable geometry of a silicon nanoantenna is a nanodisk on a dielectric substrate which can be nowadays readily fabricated by means of electron beam lithography and etching. When compared to nanospheres, nanodisks represent a body with radial symmetry but with sharp rectangular edges. For high dielectric permittivity of a nanoantenna material analytical methods appears to be inefficient and the problem can be analysed only by numerical techniques.

**Figure 11:**
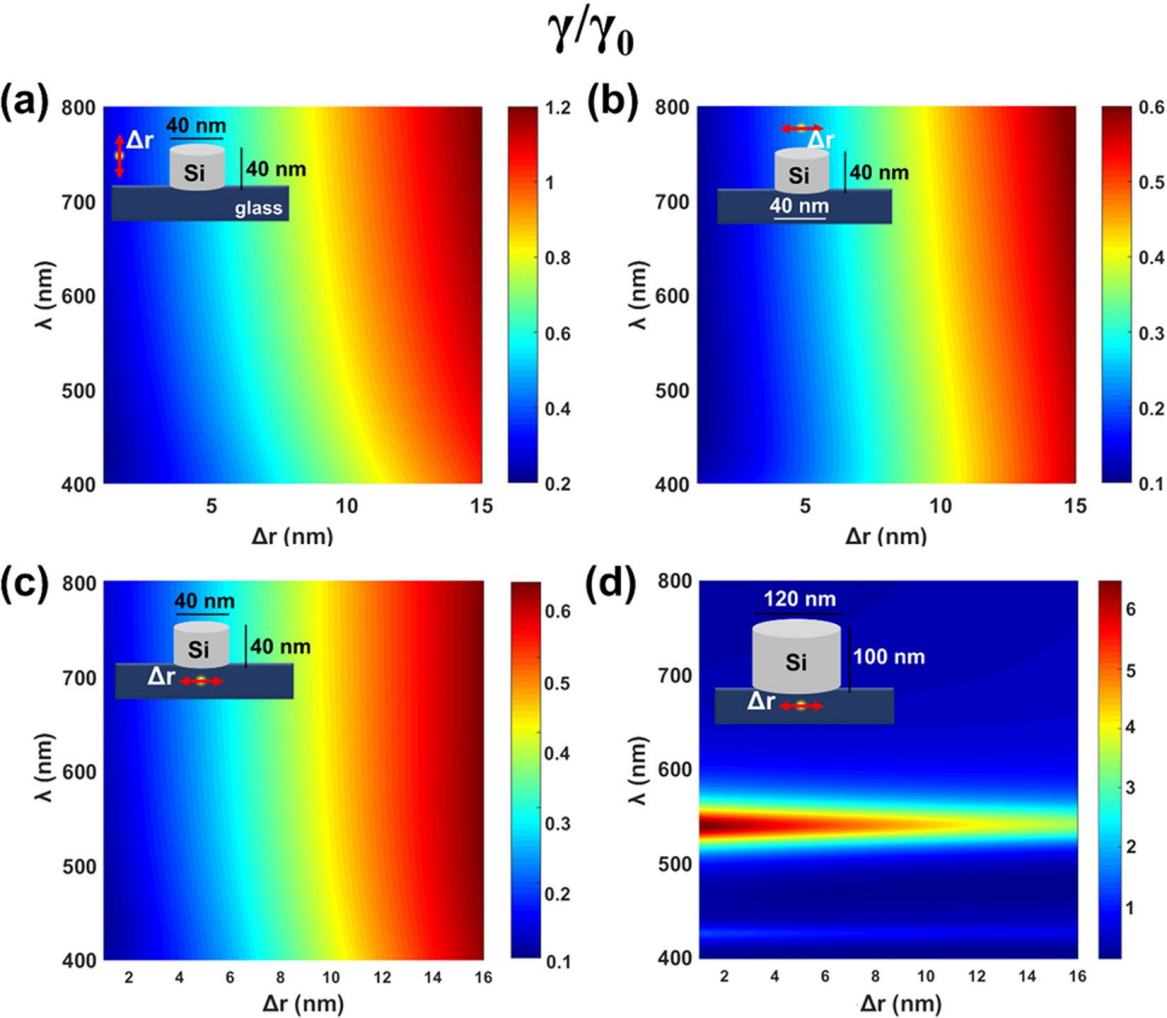
Calculated radiative decay rate γ modification for a dipole emitter near a silicon cylinder with respect to its decay rate in vacuum γ_0_ as a function of emission wavelength λ and emitter—cylinder spacing Δ*r* for the disk (**a**–**c**) diameter *d* = 40 nm and the disk height *h* = 40 nm and (**d**) disk diameter *d* = 120 nm and the disk height *h* = 100 nm. Dipole orientation and position are shown in the inserts.

**Figure 12:**
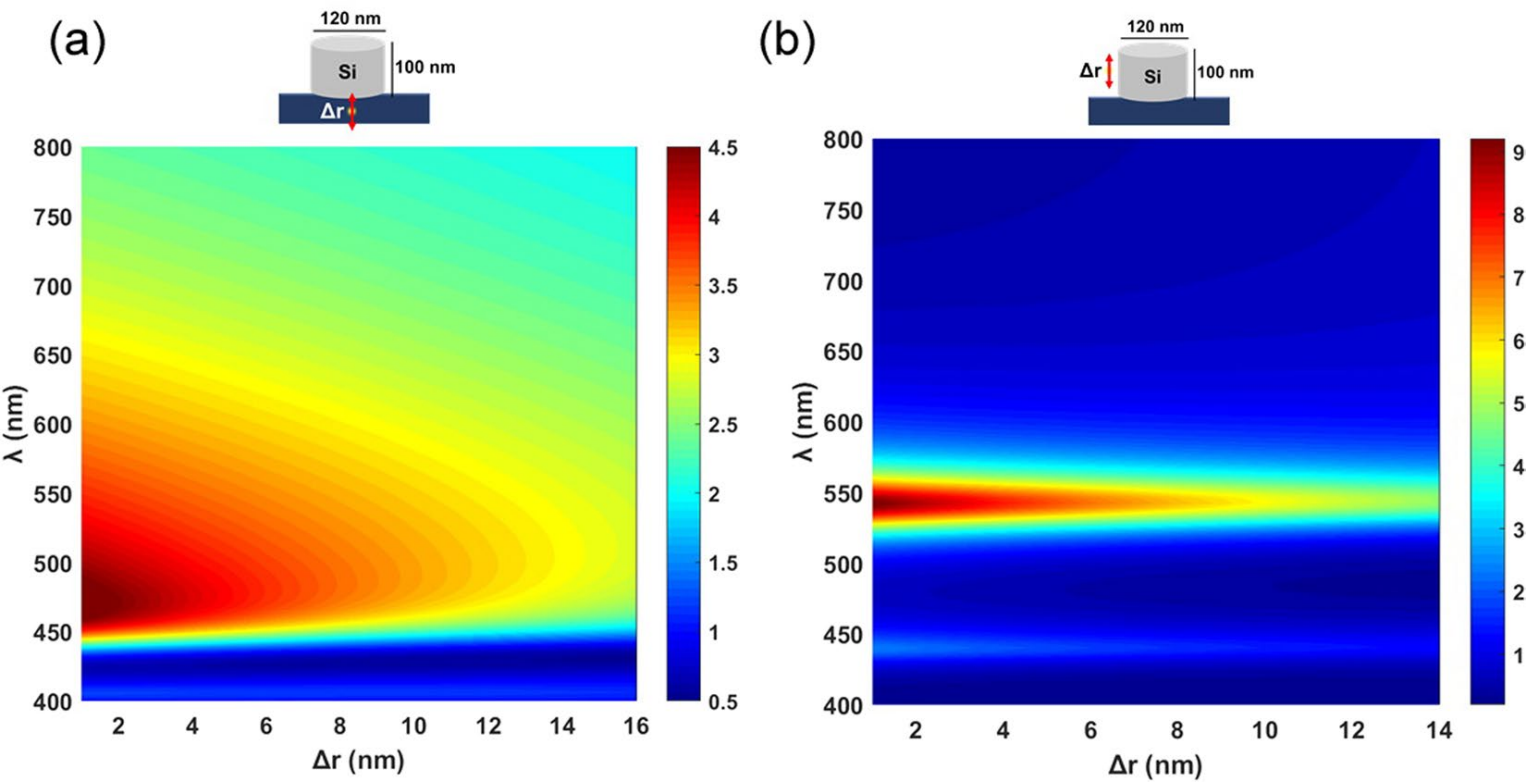
Calculated radiative decay rate γ modification for a dipole emitter near a silicon cylinder with respect to its decay rate in vacuum γ_0_ as a function of emission wavelength λ and emitter—cylinder spacing Δ*r* for the disk diameter *d* = 120 nm and the disk height *h* = 100 nm.

To check feasibility of nanodisks for inhibition of excited state decay rate, we chosed a nanodisk volume close to those explored in Fig. 9 for nanospheres. In sample calculations the height of the Si nanocylinder was set to 40 nm, diameter was 40 nm. To include substrate effects, a nanocylinder was placed on a semi-infinite glass substrate. The symmetry of the system implies three different dipole positions: near the side and near the top and bottom planes of the nanodisk. In all cases we calculated the dependence of the radiative decay rate on the distance between a dipole and a nanodisk. The results are presented in Fig. 11(a–c). The polarization in presented results was chosen accordingly to have inhibited decay rate. For an emitter oriented normally to a cylinder surface decay rate experiences enhancement for all emitter positions. Inhibition occurs for emitters oriented parallel to a disk surface, for top, bottom and side locations. For this size of the nanodisk the highest slowing down in the decay rate is of one order of the magnitude. Notably the main contribution to the effect comes from a nanodisk but the effect of a substrate/air boundary does also present in accordance with the known interface effect on radiative lifetimes^62^. We also studied the bigger Si nanodisk on the substrate (Fig. 11(d) and Fig. 12) to compare it with the results presented in Fig. 6(d). The height of the Si nanocylinder was set to 100 nm, diameter was 120 nm. For this size two enhancement resonances appear in the spectral range explored, which are associated with a magnetic and electric dipolar modes. The other part of the map is similar to the response of the Si sphere of the 100 nm diameter.

To further examine the properties of bigger disks, we show the radiaitive rate modification for the two different positions, including one in the glass substrate, and for dipole orientation parallel to the side wall of the cylinder. For the arrangement in Fig. 12(a), the bigger disk exhibits mainly enhancement rather than inhibition of decay everythere except for the deep blue and violet where minor inhibition presents. For the arrangement shown in Fig. 12(b), the situation looks similar to Fig. 11(d), i.e. a narrow spectral range corresponds to enhancement with inhibition in the violet and in the red.

One can see that comparison of the results for metal and dielectric nanoantennas shows that generally metal offers wider range of decay rate control but inevitable enhancement of nonradiative rate does not allow for pronounced inhibition of the total decay rate. This metal nanoantenna property makes its potential applications for all photochemical and photovoltaic systems pretty tricky and questionable. The dielectric counterparts, namely semiconductor nanoantennas, allow for both enhancement and inhibition of decay by one order of the magnitude which make these structures promising in photovoltaics and also in photochemistry where long living electron excited states are desirable.

## Conclusions

We pose an issue that for the wide range of photochemical and photovoltaic processes excited state decay inhibition rather than enhancement becomes a matter of concern. In the context of bioinspired photovoltaics based on photosynthesis elements, the lower excited state of a chlorophyll molecule in a reaction center should be long-living no matter whether the final quantum yield of luminescence is high or not, and the chlorophyll luminescence in these implementations should remain beyond the quest. For different types of chlorophyll the lowest excited state corresponds to the photon energy in the spectral rage of approximately 600–800 nm and in this work we examined theoretically the possibility to “freeze” an excited state using metal and dielectric (semiconductor) nanoantennas. For the simplest and experimentally affordable case of colloidal spherical nanoparticles we found that silver nanoparticles promise approximately 2-fold decay slowing down as compared to that in vacuum by means of simultaneous 100-fold inhibition of radiative decay and similar enhancement of non-radiative one. This 2-fold inhibition needs a dipole alignment parallel to a sphere surface, and can be only slightly improved towards 2.5 times inhibition by using a thin dielectric shell over a metal particle surface. Contrary to a metal nanoantenna, the dielectric one based on silicon promises more than one order of the magnitude inhibition of the decay but without bringing non-radiative losses promoting an additional decay path. However, inhibition occurs only for a dipole moment parallel to the sphere surface, as was in the case with metal antennas. Thus adsorption specificity, surface functionalization and/or chemical linking should be applied for the desirable dipole orientation. Additionally, we found that silicon nanoantennas offer more than one order of the magnitude enhancement of radiative decay within the visible for the dipole orientation normal to the sphere surface, and approximately 2-fold enhancement under condition of randomly oriented dipoles. Thus dielectric (semiconductor) nanoantennas pave a way to low-lossy control of excited states including both enhancement and inhibition of decay, the latter being of importance in bioinspired photovoltaics.

## Methods

We consider the simplest case of a single solid spherical nanobody as a nanoantenna to control the excited state decay rate. The calculation scheme has been described elsewhere^21,63^. The radiative *γ*_*rad*_ and the total *γ*_*tot*_ decay rates for an emitter can be calculated as

3 $$\begin{array}{rcl}{\frac{{\gamma }_{{rad}}}{{\gamma }_{0}}|}_{P} & = & \frac{3}{2}\sum _{m=1}^{\infty }m(m+1)\,(2m+1){|\frac{{\psi }_{m}({k}_{0}{r}_{0}n)}{{({k}_{0}{r}_{0}n)}^{2}}+{A}_{m}\frac{{\zeta }_{m}({k}_{0}{r}_{0}n)}{{({k}_{0}{r}_{0}n)}^{2}}|}^{2},\\ {\frac{{\gamma }_{tot}}{{\gamma }_{0}}|}_{P} & = & 1+\frac{3}{2}\sum _{m=1}^{\infty }m(m+1)\,(2m+1)\mathrm{Re}\{{A}_{m}{(\frac{{\zeta }_{m}({k}_{0}{r}_{0}n)}{{({k}_{0}{r}_{0}n)}^{2}})}^{2}\},\end{array}$$

for the emitter dipole moment oriented perpendicular (normally) to the sphere surface, denoted by the ‘P’ subscript, and

4 $$\begin{array}{ccc}{\frac{{\gamma }_{rad}}{{\gamma }_{0}}|}_{T} & = & \frac{3}{4}\sum _{m=1}^{{\rm{\infty }}}(2m+1)({|\frac{{\psi }_{m}({k}_{0}{r}_{0}n)}{{k}_{0}{r}_{0}n}+{B}_{m}\frac{{\zeta }_{m}({k}_{0}{r}_{0}n)}{{k}_{0}{r}_{0}n}|}^{2}\\  &  & +\,{|\frac{{{\psi }^{{\rm{^{\prime} }}}}_{m}({k}_{0}{r}_{0}n)}{{k}_{0}{r}_{0}n}+{A}_{m}\frac{{{\zeta }^{{\rm{^{\prime} }}}}_{m}({k}_{0}{r}_{0}n)}{{k}_{0}{r}_{0}n}|}^{2})\\ {\frac{{\gamma }_{tot}}{{\gamma }_{0}}|}_{T} & = & 1+\frac{3}{4}\sum _{m=1}^{{\rm{\infty }}}(2m+1){\rm{R}}{\rm{e}}\{{B}_{m}{(\frac{{\zeta }_{m}({k}_{0}{r}_{0}n)}{{k}_{0}{r}_{0}n})}^{2}+{A}_{m}{(\frac{{{\zeta }^{{\rm{^{\prime} }}}}_{m}({k}_{0}{r}_{0}n)}{{k}_{0}{r}_{0}n})}^{2}\},\end{array}$$

for the emitter dipole moment oriented tangentially (i.e. parallel) to the sphere surface, denoted by the ‘T’ subscript. In Eqs (3), (4) *γ*_0_ is decay rate in a free space without a nanobody, ‘Re’ means the real part of the expression, ${\psi }_{m}(x)=x{j}_{m}(x)$ and ${\zeta }_{m}(x)=x{h}_{m}^{(1)}(x)$ are Ricatti–Bessel functions, *j*_*n*_(*x*) and ${h}_{n}^{(1)}(x)$ are the spherical Bessel functions^64^, *k*_0_ is wave number in vacuum, *r*_0_ = *a* + Δ*r* is the distance from a nanoparticle center to an emitter, *a* is the radius of a spherical nanoparticle, *n* is the refractive index of ambient medium, and

5 $$\begin{array}{rcl}{A}_{m} & = & -\,(\frac{\sqrt{\varepsilon }{\psi }_{m}({k}_{0}a\sqrt{\varepsilon }){\psi ^{\prime} }_{m}({k}_{0}an)-n{\psi ^{\prime} }_{m}({k}_{0}a\sqrt{\varepsilon }){\psi }_{m}({k}_{0}an)}{\sqrt{\varepsilon }{\psi }_{m}({k}_{0}a\sqrt{\varepsilon }){\zeta ^{\prime} }_{m}({k}_{0}an)-n{\psi ^{\prime} }_{m}({k}_{0}a\sqrt{\varepsilon }){\zeta }_{m}({k}_{0}an)}),\\ {B}_{m} & = & -\,(\frac{n{\psi }_{m}({k}_{0}a\sqrt{\varepsilon }){\psi ^{\prime} }_{m}({k}_{0}an)-\sqrt{\varepsilon }{\psi ^{\prime} }_{m}({k}_{0}a\sqrt{\varepsilon }){\psi }_{m}({k}_{0}an)}{n{\psi }_{m}({k}_{0}a\sqrt{\varepsilon }){\zeta ^{\prime} }_{m}({k}_{0}an)-\sqrt{\varepsilon }{\psi ^{\prime} }_{m}({k}_{0}a\sqrt{\varepsilon }){\zeta }_{m}({k}_{0}an)}),\end{array}$$

are the Mie coefficients for the field reflected from a nanoparticle surface^65^, primes denote derivatives, and *ε* is the nanoparticle complex dielectric permittivity.

The total decay rate near a metal body typically higher than the radiative one because of the contribution from the non-radiative counterpart *γ*_*nonrad*_, resulting from Joule losses in a metal body, i. e. *γ*_*tot*_ = *γ*_*rad*_ + *γ*_*nonrad*_. For a dielectric nanobody the imaginary part of dielectric permittivity *ε* can be neglected in many practical cases, then total and radiative rates coincide, i. e. *γ*_*rad*_ = *γ*_*tot*_ = *γ* holds.

In the case of averaging over dipole moment orientations, the decay rates (labeled with an ‘A’ subscript) read,

6 $${\frac{{\gamma }_{{rad}}}{{\gamma }_{0}}|}_{A}=\frac{1}{3}({\frac{{\gamma }_{{rad}}}{{\gamma }_{0}}|}_{P})+\frac{2}{3}({\frac{{\gamma }_{{rad}}}{{\gamma }_{0}}|}_{T}),\,{\frac{{\gamma }_{{tot}}}{{\gamma }_{0}}|}_{A}=\frac{1}{3}({\frac{{\gamma }_{{tot}}}{{\gamma }_{0}}|}_{P})+\frac{2}{3}({\frac{{\gamma }_{{tot}}}{{\gamma }_{0}}|}_{T}).$$

Finally, in the case of a spherical nanoparticle with a dielectric shell with *h* thickness of material with refractive index *n*_*s*_, the Mie coefficients *A*_*m*_*,B*_*m*_ should be replaced^66^,

7 $$\begin{array}{rcl}{A}_{m}\to {C}_{m} & = & -\,(\frac{{n}_{s}{{\rm{\Phi }}}_{m}({k}_{0}b{n}_{s}){\psi ^{\prime} }_{m}({k}_{0}bn)-n{{\rm{\Phi }}^{\prime} }_{m}({k}_{0}b{n}_{s}){\psi }_{m}({k}_{0}bn)}{{n}_{s}{{\rm{\Phi }}}_{m}({k}_{0}b{n}_{s}){\zeta ^{\prime} }_{m}({k}_{0}bn)-n{{\rm{\Phi }}^{\prime} }_{m}({k}_{0}b{n}_{s}){\zeta }_{m}({k}_{0}bn)}),\\ {B}_{m}\to {D}_{m} & = & -\,(\frac{n{{\rm{\Psi }}}_{m}({k}_{0}b{n}_{s}){\psi ^{\prime} }_{m}({k}_{0}bn)-{n}_{s}{{\rm{\Psi }}^{\prime} }_{m}({k}_{0}b{n}_{s}){\psi }_{m}({k}_{0}bn)}{n{{\rm{\Psi }}}_{m}({k}_{0}b{n}_{s}){\zeta ^{\prime} }_{m}({k}_{0}bn)-{n}_{s}{{\rm{\Psi }}^{\prime} }_{m}({k}_{0}b{n}_{s}){\zeta }_{m}({k}_{0}bn)}),\end{array}$$

where *b* = *a* + *h* is the external nanosphere radius, and other notations read

8 $${{\rm{\Phi }}}_{m}(x)={\psi }_{m}(x)+{\alpha }_{m}{\zeta }_{m}(x),\,{{\rm{\Psi }}}_{m}(x)={\psi }_{m}(x)+{\beta }_{m}{\zeta }_{m}(x),$$

with

9 $$\begin{array}{rcl}{\alpha }_{m} & = & -(\frac{\sqrt{\varepsilon }{\psi }_{m}({k}_{0}a\sqrt{\varepsilon }){\psi ^{\prime} }_{m}({k}_{0}a{n}_{s})-{n}_{s}{\psi ^{\prime} }_{m}({k}_{0}a\sqrt{\varepsilon }){\psi }_{m}({k}_{0}a{n}_{s})}{\sqrt{\varepsilon }{\psi }_{m}({k}_{0}a\sqrt{\varepsilon }){\zeta ^{\prime} }_{m}({k}_{0}a{n}_{s})-{n}_{s}{\psi ^{\prime} }_{m}({k}_{0}a\sqrt{\varepsilon }){\zeta }_{m}({k}_{0}a{n}_{s})}),\\ {\beta }_{m} & = & -(\frac{{n}_{s}{\psi }_{m}({k}_{0}a\sqrt{\varepsilon }){\psi ^{\prime} }_{m}({k}_{0}a{n}_{s})-\sqrt{\varepsilon }{\psi ^{\prime} }_{m}({k}_{0}a\sqrt{\varepsilon }){\psi }_{m}({k}_{0}a{n}_{s})}{{n}_{s}{\psi }_{m}({k}_{0}a\sqrt{\varepsilon }){\zeta ^{\prime} }_{m}({k}_{0}a{n}_{s})-\sqrt{\varepsilon }{\psi ^{\prime} }_{m}({k}_{0}a\sqrt{\varepsilon }){\zeta }_{m}({k}_{0}a{n}_{s})}).\end{array}$$

The numerical simulations for nanodisks were performed with a help of a commercial software from Lumerical, based on the Finite-difference time-domain method^67^. We use perfect match layer boundary conditions^68^. In all calculations, the data on metal and semiconductor dielectric functions were taken from^69,70^.

The radiation power for an emitter can be found by integration of the normal component of Poynting vector over closed surface for *r* → ∞, in this case equations can be reduced to the more simple for m. In spherical coordinates one has,

10 $$\begin{array}{ccc}{P}_{rad} & = & \frac{c}{8\pi }{\rm{R}}{\rm{e}}{\int }_{0}^{2\pi }d\phi {\int }_{0}^{\pi }d\theta \,\sin \,\theta {r}^{2}{([{{\bf{E}}}_{tot}\times {{\bf{H}}}_{tot}^{\ast }]\cdot {\bf{n}})|}_{r\to {\rm{\infty }}},\\ {P}_{0} & = & \frac{c}{8\pi }{\rm{R}}{\rm{e}}{\int }_{0}^{2\pi }d\phi {\int }_{0}^{\pi }d\theta \,\sin \,\theta {{r}^{2}([{{\bf{E}}}_{0}\times {{\bf{H}}}_{0}^{\ast }]\cdot {\bf{n}})|}_{r\to {\rm{\infty }}}=\frac{c{k}_{0}^{4}}{3}{|{{\bf{d}}}_{0}|}^{2},\end{array}$$

where **E**_*tot*_ = **E**_*refl*_ + **E**_0_ and **H**_*tot*_ = **H**_*refl*_ + **H**_0_ are the full electric and magnetic fields, respectively, **E**_*refl*_ and **H**_*refl*_ are reflected fields, **E**_0_, **H**_0_ are source fields. Here *с* is speed of light in vacuum, and **n** is the unit vector normal to the sphere surface. Square brackets [×] denote vector product and the ordinary brackets (·) denote scalar product. The *D*(Ω) function is then calculated as

11 $$D({\rm{\Omega }})={\frac{3{r}^{2}\mathrm{Re}([{{\bf{E}}}_{{tot}}\times {{\bf{H}}}_{{tot}}^{\ast }]\cdot {\bf{n}})}{8\pi {k}_{0}^{4}{|{{\bf{d}}}_{0}|}^{2}}|}_{r\to \infty }.$$

## Data Availability

All data reported are available upon request to the corresponding author.
